# Omega-3 Polyunsaturated Fatty Acids Supplementation Alleviate Anxiety Rather Than Depressive Symptoms Among First-Diagnosed, Drug-Naïve Major Depressive Disorder Patients: A Randomized Clinical Trial

**DOI:** 10.3389/fnut.2022.876152

**Published:** 2022-07-12

**Authors:** Rong Yang, Lu Wang, Kun Jin, Song Cao, Chujun Wu, Jimin Guo, Jindong Chen, Hui Tang, Mimi Tang

**Affiliations:** ^1^Department of Pharmacy, Xiangya Hospital, Central South University, Changsha, China; ^2^National Clinical Research Center for Geriatric Disorders, Xiangya Hospital, Central South University, Changsha, China; ^3^Department of Psychiatry, National Clinical Research Center for Mental Disorders, China National Technology Institute on Mental Disorders, The Second Xiangya Hospital of Central South University, Changsha, China; ^4^Department of Microbiology, Immunology and Molecular Genetics, University of California, Los Angeles, Los Angeles, CA, United States

**Keywords:** nutrient intervention, antidepressant, omega-3 polyunsaturated fatty acids, major depressive disorders, childhood abuse, social support

## Abstract

**Background:**

Omega-3 polyunsaturated fatty acids (n-3 PUFAs) augmentation of antidepressants has shown great potential in the prevention and treatment of major depressive disorders (MDD).

**Objective:**

To investigate the effect of n-3 PUFAs plus venlafaxine in patients with first-diagnosed, drug-naïve depression.

**Method:**

A total of 72 outpatients with first-diagnosed depression were recruited. The daily dose of 2.4 g/day n-3 PUFAs or placebo plus venlafaxine was used for over 12 weeks. The outcomes were assessed by the Hamilton depression scale (HAMD), Hamilton anxiety scale (HAMA), Beck depression inventory (BDI), and Self-rating anxiety scale (SAS).

**Results:**

Both groups exhibited improvement on clinical characteristics at week 4 and week 12 compared with baseline. The rate of responders for anxiety in n-3 PUFAs group (44.44%) was significantly higher than that in placebo group (21.21%) at week 4 (χ^2^ = 4.182, *p* = 0.041), while week 12 did not show a difference (χ^2^ = 0.900, *p* = 0.343). The rate of responders for depression at both week 4 (χ^2^ = 0.261, *p* = 0.609) and week 12 (χ^2^ = 1.443, *p* = 0.230) showed no significant difference between two groups. Further analysis found that Childhood Trauma Questionnaire (CTQ) had positive correlation with HAMA (*r* = 0.301, *p* = 0.012), SAS (*r* = 0.246, *p* = 0.015), HAMD (*r* = 0.252, *p* = 0.038) and BDI (*r* = 0.233, *p* = 0.022) with Pearson correlation analysis. Social Support Rating Scale (SSRS) had negative correlation with SAS (*r* = −0.244, *p* = 0.015) and BDI (*r* = −0.365, *p* = 0.000).

**Conclusion:**

This trial found that n-3 PUFAs supplementation in favor of venlafaxine alleviated the anxiety symptoms rather than depressive symptoms at the early stage of treatment (4 weeks) for first-diagnosed, drug-naïve depressed patients. However, the advantage disappeared in long-term treatment. Furthermore, childhood abuse and social support are closely related to the clinical and biological characteristics of depression. Both childhood trauma and lack of social support might be predictors of poor prognosis in depression.

**Clinical Trial Registration:**

[clinicaltrials.gov], identifier [NCT03295708].

## Introduction

Depression is a common and serious mental disorder affecting more than 264 million people in the world (1). The World Health Organization reported that only 50% of severe cases have received effective treatment in developed countries, and less than 25% in some developing countries due to high prevalence and not recognized by professionals (2). Among them, only 50−70% of the patients respond to a single antidepressant medication (3, 4). These low treatment percentages and low therapeutic efficacy encourage other more acceptable alternatives or augmentation treatments for depression. Nutritional intervention is acceptable while the effective strategy has attracted interest in the prevention or treatment of depression (5, 6). Several clinical studies have shown that Omega-3 polyunsaturated fatty acids (n-3 PUFAs) could promise in alleviating anxiety, depression, and other related symptoms among those with depressive disorder (7, 8). The American Psychiatric Association (APA) practice guideline also recommended n-3 PUFAs as adjunctive therapy for mood disorders, such as depression (9).

N-3 PUFAs, mainly consisting of eicosapentaenoic acid (EPA) and docosahexaenoic acid (DHA) (10, 11), are essential and critical for the maintenance of neuronal membrane structure, modulating inflammatory responses, mediating signal transduction, affecting the release and function of serotonin and dopamine in the nervous system (6, 12–15). Generally speaking, the physiological functions of EPA and DHA are different: EPA mainly plays an anti-inflammatory role in depression, while DHA mainly maintains cell membrane integrity and fluidity (16). These biological actions of n-3 PUFAs may influence the development of mental disorders, such as depression. Some clinical studies confirmed that a higher risk of mood disorder was associated with lower plasma levels of N-3 PUFA (17, 18), therefore, most studies have explored the efficacy of EPA, DHA, or their mixtures in MDD (19, 20). Due to the heterogeneity of these trials, the results are controversial and inconclusive (8, 21). The discrepant findings might be affected by the dosage and proportion of n-3 PUFAs, the treatment duration, and the enrolment of different types of participants (22, 23). EPA-major formulations and a daily dose of over 1.5 g of N-3 PUFA demonstrated clinical benefits in depression (24–27). Furthermore, the severity of clinical symptoms may affect the efficacy of N-3 PUFA. N-3 PUFA may have little or no effect on reducing the risk of depression or anxiety in mentally health participants at baseline (28). Appleton et al. also found that n-3 PUFAs may have a more pronounced effect in patients with more severe depressive symptoms (29). A growing number of clinical and, particularly, basic research has revealed the potential of N-3 PUFA in reducing depressive symptoms and the risk of onset or recurrence in MDD, yet the quality of evidence is too low to draw firm conclusions (28, 30). To summarize, there is insufficient high-quality evidence to assess the efficacy of N-3 PUFA as a treatment for depression, and more appropriate and well-designed studies will be required in the future to strengthen the clinical evidence.

Venlafaxine as the first-line drug for depression was introduced (31). Venlafaxine plus with n-3 PUFAs or placebo was investigated on the first-diagnosed, drug-naïve depressed patients for 12 weeks. Only first-diagnosed, drug-naïve depressed patients who never received any medications and psychotherapies before were recruited. It would be helpful of minimizing the heterogeneity of the participants. Interestingly, the baseline childhood trauma and social support may be the risk factors for depression and also could affect the efficacy of antidepressants during different treatment cycles (32–34). Therefore, the Childhood Trauma Questionnaire (CTQ) and Social Support Rating Scale (SSRS), which can be used for evaluation of childhood trauma and social support, respectively, were also collected. The purpose of this study was to investigate whether n-3 PUFAs could enhance the therapeutic efficacy of venlafaxine to attenuate depressive and anxiety symptoms.

## Materials and Methods

### Subjects

A total of 72 first-diagnosed, drug-naïve depressed patients aged between 18 and 45 years were included in the study from March 1st, 2017, to January 20th, 2020 in the Second Xiangya Hospital of Central South University, China. The participants must meet the Diagnostic and Statistical Manual of Mental Disorders-Fourth Edition (DSM-IV) criteria for current major depression by Structured Clinical Interview for DSM Disorders (SCID) and have a 24 item Hamilton Depression Rating Scale (HAMD) score greater than 20. The diagnoses of one patient were performed by two experienced psychiatrists. Venlafaxine was given as a therapeutic drug. The participants were excluded from the participants if they meet the following criteria: (1) suffering from other serious somatic diseases or comorbidities; (2) had serious nervous system disease; (3) in accordance with diagnostic standards of other mental illnesses; (4) were taking benzodiazepine every day, and need to be treated by electroconvulsive therapy currently or have received electroconvulsive therapy within the previous 6 months; (5) were pregnant women or lactating women; (6) had an apparent suicide attempt or suicidal behavior; (7) were taking omega-3 fatty acids supplements and eating more than 2 fish meals per week; and (8) any condition or medicines that may have an effect on biomarkers (within 1 week of the screening period or during the whole trial period) (35).

### Study Design

The study was designed as a randomized, double-blind, placebo-controlled clinical trial to test the efficiency of n-3 PUFAs as an augmentation therapy to venlafaxine over 12 weeks. After meeting eligibility criteria, participants were randomized to receive either A or B drugs by an independent pharmacist who dispensed either active or placebo capsules according to the random number table generated randomization list. The information on the A and B drugs were sealed in an envelope which was made and kept by another independent pharmacist. The information inside the envelope was not made public until the end of the experiment. All participants, psychiatrists, and the researchers who met with the participants were blinded to group allocation. All participants needed to take venlafaxine (instruction recommend a dosage range of 75-225 mg/day) throughout the trial. The daily dose of n-3 PUFAs was 8 * 1 g capsules (EPA 1440 mg/day, DHA 960 mg/day) per day for 12 weeks based on the reported studies (12, 36, 37). The placebo group was given 8 * 1 g soybean oil placebo capsules daily. Soybean oil placebo capsules contained soybean oil with 1% fish oil so that it was flavored to taste and smell similar to the fish oil capsules. Both fish oil capsules and placebo capsules were supplied by Hunan Kang Q. Y. Bai Biological Technology Co., Ltd.

Following screening and baseline visits, study participants visit at week 4 and week 12. The 12 weeks of treatment length was determined based on previous trials of n-3 PUFAs for the treatment of depression (38–41). During the three follow-up visits (at baseline, week 4 and week 12) and two telephone contacts at the beginning of week 4 and week 12, the investigator encouraged medication adherence. The remaining medications were counted at each post-treatment follow-ups and subtracted from the number provided to determine adherence. Moreover, erythrocyte membrane EPA and DHA levels were obtained at baseline and post-treatment to confirm adherence to the fish oil capsules in the intervention arm. This study was approved by the ethics committee of Second Xiangya Hospital (MDD201610), and written informed consent was obtained from all participants.

### Clinical Assessment

The baseline data included related demographics, clinical evaluations of psychiatric symptoms, a comprehensive medical history, a physical measurement of weight and height, and laboratory tests, including levels of EPA, DHA, and n-3 PUFAs. Follow-up visits were conducted at weeks 4 and 12 after the enrollment and drug treatment. The primary outcome was the change in 24 item Hamilton Depression Rating Scale (HAMD), and the secondary outcome was the change in the Hamilton anxiety scale (HAMA). Two self-reported rating scales were also introduced in this study, which was Beck depression inventory (BDI) and the Self-rating anxiety scale (SAS). All four questionnaires were completed by participants at each visit. The change in HAMD and HAMA total score from baseline at week 4 and week 12 were the response criterion. Responders were identified as those with a 50% or greater decrease in HAMD or HAMA score as previously described (42, 43). While remission was defined as the absolute value of HAMD or HAMA score less than 8.

The CTQ score was evaluated because of high internal consistency and strong retest reliability (44, 45). SSRS has good reliability and validity and is an important clinical assessment for depressed patients. CTQ and SSRS were completed at baseline by all participants. The receiver operating curve (ROC) was used to analyze the relationships between therapeutic effect and childhood trauma or social support. The sensitivity and specificity of clinical findings related to CTQ and SSRS were expressed by ROC analysis. The area under the curve (AUC) was calculated for each plot and used to evaluate the value of CTQ and SSRS in predicting the prognosis of depressed patients. Sensitivity and specificity were calculated by the following formulas (46):


$$\begin{array}{c} \mathrm{Sensitivity}\\ =\mathrm{True}\mathrm{positives}/\left(\mathrm{True}\mathrm{positives}+\mathrm{False}\mathrm{negatives}\right)\times 100 \end{array}$$

$$\begin{array}{c} \mathrm{Specificity}\\ =\mathrm{True}\mathrm{negatives}/\left(\mathrm{True}\mathrm{negatives}+\mathrm{False}\mathrm{positives}\right)\times 100 \end{array}$$

### Fatty Acid Determination

Fatty acid determination followed the method from our previous report with a slight modification. Briefly, blood collection was performed in the early morning at baseline, 4 weeks and 12 weeks of antidepressant treatment for participants. The fasting blood samples were drawn into 5-ml vacutainer tubes containing EDTA, and then the blood was centrifuged for 5 min (3000 r/min). The red blood cells in the lower layer were transferred into Eppendorf tubes and stored at −80°C until lipid extraction and GC/MS analysis. To extract lipid, erythrocyte membranes were isolated from 150 μl of red blood cells vortexed with ice water for 3 min. Isopropanol (1.5 ml) and dichloromethane (1 ml) were successively added to the erythrocyte membranes. Then, 1 ml of 0.1 M potassium chloride solution was added to the mixture. The mixture was blended for 3 min, and centrifuged for 5 min (2,000 rpm/min). The upper phase was removed, and we repeated the previous step on the lower layer twice. Subsequently, 10 μl of 100 μM butylated hydroxytoluene was added to the organic layer and the mixture was evaporated to dryness by nitrogen. The residue was taken through the same processing and analysis method as previously reported (11). The concentrations of fatty acids (ng/ml packed red blood cell) were expressed as the mean ± standard deviation (SD).

#### Statistical Analysis

Continuous variables were expressed as the mean ± standard deviation (SD) and categorical variables were presented as numbers. The difference between the mean values for each variable among the n-3 PUFAs augmentation group and placebo control group were tested for significance using an independent t test or paired t test (continuous variables). Categorical variables analysis was assessed using the χ^2^ test. The Generalized Estimating Equation analysis was used for rating scales that repeatedly evaluated at each visit. Correlation of the DHA, EPA, total n-3 PUFAs level, and CTQ score with symptomatic index in the depression patients were calculated using Pearson’s correlation, and Bonferroni corrections were utilized to adjust for multiple testing. All statistical analyses were carried out using SPSS statistical software version 24.0. The *p*-value of 0.05 was used to determine the statistical difference. We calculated the sample size by the non-inferiority test of the comparison of the two groups (*a* = 0.05, β = 0.02) in PASS 15, and 30% attrition was taken into account. With a sample size of 21 (treatment group) and 27 (control group), the trial will have more than 80% power to detect a difference.

## Results

### Study Sample

**FIGURE 1:**
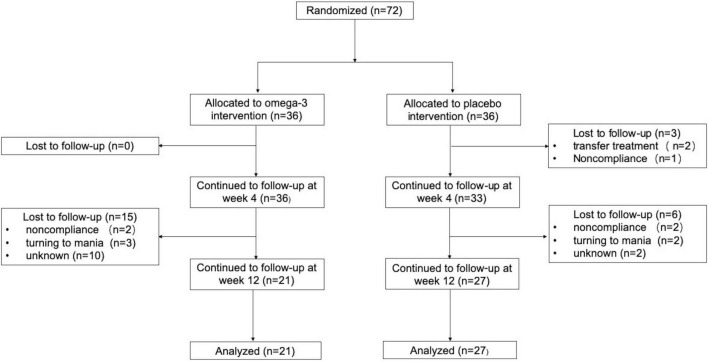
Flow of participants in the Trial. Non-compliance includes being unwilling to take medicine and taking medicine irregularly.

A total of 72 first-diagnosed, drug-naïve depressed patients met all the study’s eligibility criteria and randomly assigned to venlafaxine with n-3 PUFAs augmentation intervention group (*n* = 36; 50%) or venlafaxine with the placebo group (*n* = 36; 50%). The baseline characteristics of both groups were no statistical differences (*p* ≥ 0.05). The detailed data were listed in Table 1. Of the 72 participants, 69 participants completed the 4-week follow-up: 36 participants in the n-3 PUFAs intervention group and 33 participants in the placebo group continued the 4-week trial. Finally, 49 individuals completed the trial at week 12: 21 participants in the n-3 PUFAs group and 27 participants in the placebo group finished the 12-week follow-up. The detailed flow of participants in the trial is shown in Figure 1.

**TABLE 1:** Baseline characteristics.

Parameter	n-3 PUFAs (*n* = 36)	Placebo (*n* = 36)	t/χ 2	*P*-value
Age (y), mean (SD)	26.33 (8.07)	27.11 (8.14)	−0.407	0.685
Males/females	15/21	11/25	0.963	0.326
BMI (kg/m^2^)	20.58 (3.02)	21.83 (2.88)	0.006	0.937
Education (years)	14.42 (2.94)	13.26 (3.21)	1.566	0.122
Source of participants			0.229	0.633
Urban	22 (52.38%)	20 (47.62%)		
Rural	14 (46.67%)	16 (53.33%)		
Baseline DHA levels (umol/L), mean (SD)	0.76 (0.43)	0.81 (0.54)	−0.478	0.634
Baseline EPA levels (umol/L), mean (SD)	0.05 (0.03)	0.06 (0.03)	−0.506	0.615
Baseline Omega-3 fatty acids (umol/L), mean (SD)	1.29 (0.71)	1.41 (0.83)	−0.668	0.507
Baseline CTQ score, mean (SD)	52.61 (15.70)	52.17 (13.88)	0.125	0.901
Emotional abuse, mean (SD)	9.89 (4.52)	9.14 (3.77)	0.761	0.449
Physical abuse, mean (SD)	7.00 (3.14)	6.71 (2.47)	0.427	0.671
Sexual abuse, mean (SD)	6.05 (1.70)	6.29 (2.05)	−0.523	0.603
Emotional neglect, mean (SD)	13.65 (5.34)	12.80 (5.17)	−0.684	0.496
Physical neglect, mean (SD)	10.27 (3.13)	10.54 (4.08)	−0.319	0.751
SSRS, mean (SD)	28.11 (8.09)	28.83 (5.41)	−0.445	0.657
Subjective support	15.95 (4.95)	15.86 (4.29)	0.078	0.938
Objective support	7.22 (1.87)	7.22 (3.45)	−0.009	0.993

*n-3 PUFAs, Omega-3 polyunsaturated fatty acids; BMI, Body Mass Index; DHA, docosahexaenoic acid; EPA, eicosapentaenoic acid; CTQ, Childhood Trauma Questionnaire; SSRS, Social Support Rate Scale.*

### Efficacy Outcomes

**FIGURE 2:**
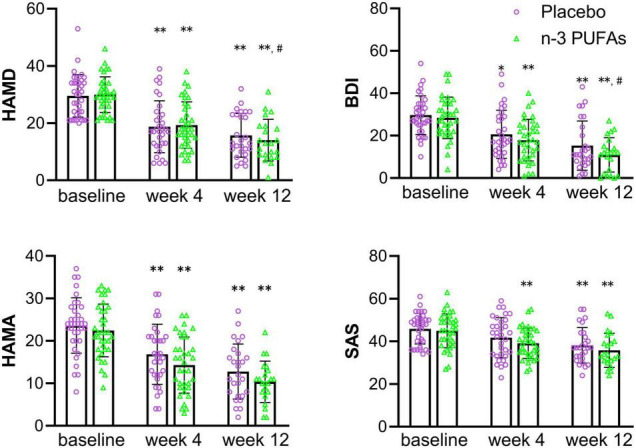
The scores on depression and anxiety questionnaires in n-3 PUFAs and placebo group. *significantly different compared with baseline, # significantly different compared with week 4, *p < 0.05, ^**^p < 0.01, # *p* < 0.05, ## *p* < 0.01. n-3 PUFAs, Omega-3 polyunsaturated fatty acids; HAMD, Hamilton Depression Scale; BDI, Beck Depression Inventory; HAMA, Hamilton anxiety scale; SAS, Self-rating Anxiety Scale. For n-3 PUFAs group, *n* = 36, after week 4, *n* = 36, and after week 12, *n* = 21. For the placebo group, *n* = 36, after week 4, *n* = 33, and after week 12, *n* = 27.

Results of changes in depression and anxiety questionnaire scores at baseline and after week 4 and week 12 are displayed in Figure 2 and Table 2. The HAMD scores in n-3 PUFAs group and placebo group at week 4 and week 12 were reduced compared with baseline (*p* < 0.01). The patients who took n-3 PUFAs combined with venlafaxine had lower HAMD scores at week 12 than week 4 (*p* < 0.01). BDI scores both at week 4 and week 12 (*p* < 0.01) were lower than baseline in both the n-3 PUFAs group and the placebo group. Lower BDI scores were seen at week 12 than week 4 in the n-3 PUFAs group (*p* < 0.01) and placebo group (*p* < 0.05). HAMA anxiety questionnaire scores had a similar decreasing trend to BDI scores. The subjects in n-3 PUFAs and placebo groups get lower HAMA scores at week 4 and week 12 compared with baseline (*p* < 0.01). N-3 PUFAs group (*p* < 0.01) and placebo group (*p* < 0.05) had lower HAMA scores at week 12 than week 4. The patients in the n-3 PUFAs group had lower SAS scores at week 4 and week 12 than baseline (*p* < 0.01).

**TABLE 2:** The scores on depression and anxiety questionnaires in n-3 PUFAs and placebo group.

	Baseline	Week 4	Week 12	Group Wald χ 2 (*P*-value)	Time Wald χ 2 (*P*-value)	Group * Time Wald χ 2 (*P*-value)
**HAMD**						
n-3 PUFAs	29.50 (27.11,31.89)	18.76 (15.71,21.81) ^A^	15.70 (12.87,18.53) ^B^	0.023 (0.880)	180.9 (0.000)	0.985 (0.611)
Placebo	29.97 (27.97,31.97)	19.31 (16.69,21.92) ^A^	14.05 (11.07,17.02) ^B, C^			
**BDI**						
n-3 PUFAs	29.67 (26.74,32.60)	20.64 (16.83,24.44) ^A^	15.30 (10.99,19.60) ^B, c^	2.182 (0.140)	110.4 (0.000)	0.992 (0.609)
Placebo	28.42 (25.27,31.56)	17.86 (14.70,21.02) ^A^	10.91 (7.60,14.22) ^B, C^			
**HAMA**						
n-3 PUFAs	23.61 (21.51,25.71)	17.70 (14.36,21.03) ^A^	12.78 (10.38,15.18) ^B, c^	3.406 (0.065)	156.6 (0.000)	1.282 (0.527)
Placebo	22.44 (20.44,24.45)	14.31 (12.17,16.44) ^A^	10.36 (8.38,12.35) ^B, C^			
**SAS**						
n-3 PUFAs	45.92 (43.60,48.23)	40.85 (37.79,43.91) ^A^	38.15 (35.08,41.22) ^B^	1.185 (0.276)	66.5 (0.000)	0.400 (0.819)
Placebo	44.92 (42.35,47.48)	39.11 (36.82,41.41) ^A^	35.77 (32.53,39.02) ^B^			

*n-3 PUFAs, Omega-3 polyunsaturated fatty acids; DHA, docosahexaenoic acid; EPA, eicosapentaenoic acid; HAMA, Hamilton anxiety scale; SAS, Self-rating Anxiety Scale; HAMD, Hamilton Depression Scale; BDI, Beck Depression Inventory. The rating scale scores were expressed as Mean (95% Wald confidence interval).*

*Significantly difference compared Placebo and n-3 PUFAs groups; *p < 0.05; **p < 0.01.

*A (a) Significantly difference compared baseline with week 4;a:p < 0.05;A:p < 0.01.*

*B (b) Significantly difference compared baseline with week 12;b:p < 0.05;B:p < 0.01.*

*C (c) Significantly difference compared week 4 with week 12;c:p < 0.05;C:p < 0.01.*

The rate of responders and the rate of full remission in n-3 PUFAs and placebo groups are presented in Table 3. The depression rate of responders between n-3 PUFAs and placebo group showed no significant difference at both week 4 (χ^2^ = 0.261, *p* = 0.609) and week 12 (χ^2^ = 1.443, *p* = 0.230). However, the anxiety rate of responders at week 4 in n-3 PUFAs was significantly higher than in the placebo group (χ^2^ = 4.182, *p* = 0.041). The anxiety rate of responders at week 12 had no significant difference between n-3 PUFAs group and the placebo group (χ^2^ = 0.900, *p* = 0.343). The depression rate of full remission at week 4 (χ^2^ = 0.935, *p* = 0.416), week 12 (χ^2^ = 0.003, *p* = 1.000) were no significantly different between n-3 PUFAs group and placebo group. Also, the anxiety rate of full remission at week 4 (χ^2^ = 3.892, *p* = 0.115), week 12 (χ^2^ = 0.597, *p* = 0.683) showed no significantly difference between groups.

**TABLE 3:** The number of responders and the full remission in n-3 PUFAs and placebo group.

Parameter	n-3 PUFAs		Placebo		χ 2	*P*-value
	Responders	Non-responders	Responders	Non-responders		
**The number of responders**						
**Depression**						
Week 4	11	25	12	21	0.261	0.609
Week 12	13	8	12	15	1.443	0.230
**Anxiety**						
Week 4	16	20	7	26	4.182	**0.041**
Week 12	13	8	13	14	0.900	0.343
**The number of participants who reach remission**						
**Depression**						
Week 4	2	34	4	29	0.935	0.416
Week 12	3	18	4	23	0.003	1.000
**Anxiety**						
Week 4	6	30	3	30	0.871	0.351
Week 12	3	18	4	23	0.003	0.959

*n-3 PUFAs, Omega-3 polyunsaturated fatty acids. The bold values represent the positive results.*

### Comparisons Between Depressed Patients With Childhood Trauma and Depressed Patients Without Childhood Trauma

The CTQ showed a good internal consistency (Cronbach’s α coefficient = 0.792). The data in Table 4 showed that the first-diagnosed, drug-naïve depressed patients with childhood trauma had higher baseline parameters. Depressed patients with childhood trauma had significantly higher SAS (*p* < 0.05), BDI (*p* < 0.05) scores and lower EPA (*p* < 0.05) levels than patients without childhood trauma.

**TABLE 4:** Comparisons between depressed patients with Childhood Trauma (MDD + CT) and depressed patients without Childhood Trauma (MDD).

	MDD (*n* = 20)	MDD + CT (*n* = 50)	T	*P*-value
HAMA	21.12(6.09)	24.19(6.40)	1.87	0.07
SAS	42.82(9.79)	46.90(7.67)	2.11	**0.03**
HAMD	29.04(5.09)	29.34(7.49)	−0.18	0.86
BDI	24.38(8.85)	30.35(9.05)	2.49	**0.02**
EPA	0.07(0.04)	0.05(0.03)	2.26	**0.03**
DHA	0.79(0.53)	0.85(0.59)	−0.47	0.64
Total n-3 PUFAs	1.40(0.83)	1.43(0.90)	−0.15	0.88

*Data were presented as MEAN(SD). HAMA, Hamilton anxiety scale; SAS, Self-rating Anxiety Scale; HAMD, Hamilton Depression Scale; BDI, Beck Depression Inventory; EPA, eicosapentaenoic acid; DHA, docosahexaenoic acid; n-3 PUFAs, Omega-3 polyunsaturated fatty acids. The bold values represent the positive results.*

### Correlations Between Symptomatic Index, Omega-3 Levels, and Childhood Trauma Questionnaire, Social Support Rating Scale

The data in Table 5 for first-diagnosed, drug-naïve depressed patients were analyzed separately to assess symptomatic index, n-3 PUFAs levels associated with CTQ or SSRS. Pearson correlation showed that CTQ had positive correlation with HAMA (*r* = 0.301, *p* = 0.012), SAS (*r* = 0.246, *p* = 0.015), HAMD (*r* = 0.252, *p* = 0.038) and BDI (*r* = 0.233, *p* = 0.022), and negative correlation with EPA level (*r* = −0.238, *p* = 0.029). SSRS had negative correlation with SAS (*r* = −0.244, *p* = 0.015) and BDI (*r* = −0.365, *p* = 0.00). Besides, the Pearson correlation analysis showed a symptomatic index that had no significant correlation with DHA, EPA, n-3 PUFAs levels, the detailed data were presented in Supplementary Table 1. The results indicated that some clinical symptomatic index and EPA levels would be affected by the CTQ score or SSRS score.

**TABLE 5:** Correlations between symptomatic index and n-3 PUFAs levels, CTQ, SSRS in patients with first-diagnosed drug-naïve depression.

	CTQ	SSRS
HAMA	**0.301 (0.012)**	0.043 (0.671)
SAS	**0.246 (0.015)**	−**0.244 (0.015)**
HAMD	**0.252 (0.038)**	−0.139 (0.169)
BDI	**0.233 (0.022)**	−**0.365 (0.000)**
EPA	−**0.238 (0.029)**	0.130 (0.232)
DHA	−0.114 (0.295)	0.037 (0.734)
Total n-3 PUFAs	−0.144 (0.185)	0.054 (0.620)

*HAMA, Hamilton anxiety scale; SAS, Self-rating Anxiety Scale; HAMD, Hamilton Depression Scale; BDI, Beck Depression Inventory; EPA, eicosapentaenoic acid; DHA, docosahexaenoic acid; n-3 PUFAs, Omega-3 polyunsaturated fatty acids. The bold values represent the positive results.*

### The Receiver Operating Curve Curve Analysis of Childhood Trauma Questionnaire and Social Support Rating Scale

**FIGURE 3:**
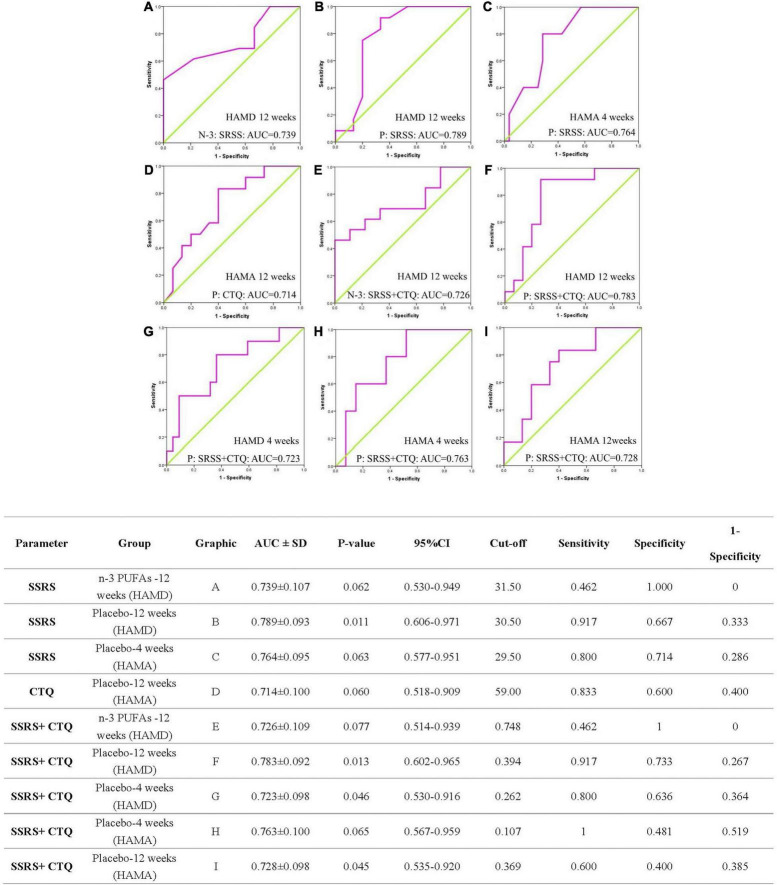
The ROC curve analysis of CTQ, SSRS, and combined effect for the prognosis of patients. ROC, receiver operating curve; AUC, area under the curve; SD, Standard deviation; CI, confidence interval; SSRS, Social Support Rate Scale; CTQ, Childhood Trauma Questionnaire; HAMA, Hamilton anxiety scale; HAMD, Hamilton Depression Scale; n-3 PUFAs, Omega-3 polyunsaturated fatty acids. The N-3 and P in images represent n-3 PUFAs and placebo group separately.

The ROC curve of CTQ, ROC curve of SSRS, and the combined ROC curve of CTQ and SSRS for the prognosis of depressed patients shown in Figure 3 and the detailed data were provided in Supplementary Tables 2–4. About the SSRS ROC curve to evaluate the responders of HAMD: for n-3 PUFAs group at week 12 the AUC was 0.739 ± 0.107 with 95% credible interval (CI) was 0.530-0.949 (*p* = 0.062); for placebo group at week 12 the AUC was 0.789 ± 0.093 (95% CI: 0.606-0.971, *p* = 0.011). The SSRS ROC curve to evaluate the responders of HAMA for placebo subjects at week 4 the AUC was 0.764 ± 0.095 (95% CI: 0.577-0.951, *p* = 0.063). The CTQ AUC to evaluate the responders of HAMA for placebo subjects at week 12 was 0.714 ± 0.100 (95% CI: 0.518-0.909, *p* = 0.060).

In view of the combined ROC curve of CTQ and SSRS to evaluate the responders of depressive symptoms, the AUC was 0.726 ± 0.109 (95%CI: 0.514-0.939, *p* = 0.077) at week 12 in n-3 PUFAs group, 0.723 ± 0.098 (95%CI: 0.530-0.916, *p* = 0.046) at week 4 in placebo and 0.783 ± 0.092 (95%CI: 0.602-0.965, *p* = 0.013) at week 12 in placebo group, respectively. The AUC of combined CTQ and SSRS ROC curve to evaluate the responders of anxiety symptoms for placebo group at week 4 was 0.763 ± 0.100 (95%CI: 0.567-0.959, *p* = 0.065) and at week 12 was 0.728 ± 0.098 (95%CI: 0.535-0.920, *p* = 0.045).

### Adverse Events

Adverse events were assessed at baseline, the 4-week and the 12-week follow-up visits. There were five participants withdrew from the trial because of turning from depression to mania. No adverse events were regarded as serious adverse events related to the study medication.

## Discussion

To our best knowledge, this is the first double-blind, placebo-controlled, randomized clinical trial to test the efficacy of n-3 PUFAs as adjunctive therapy combined with venlafaxine in the treatment of first-diagnosed, drug-naïve depressed patients. The results supported that n-3 PUFAs adjuvant therapy is more effective in improving anxiety symptoms than depressive symptoms at an early stage of treatment for depressed patients.

In this study, patients who took co-administration of venlafaxine at recommended dosage with n-3 PUFAs (EPA 1,440 mg/day, DHA 960 mg/day) or placebo had lower anxiety and depression questionnaires scores at each follow-up compared with baseline. These results were consistent with a previous study that reported that venlafaxine effectively reduced symptoms of depression in patients with MDD overall (47). We also observed that n-3 PUFAs supplementation increased the rate of responders for anxiety at week 4 and then the difference disappeared at week 12, which indicated that n-3 PUFAs supplementation could help to alleviate anxiety symptoms at an early stage of treatment for depressed patients. Considering the fact that conventional antidepressants take weeks or even months to have their full therapeutic effects (the effect varies from person to person) (48, 49), this rapid relief effect on anxiety may increase the confidence of patients and further increase treatment compliance. Furthermore, n-3 PUFAs are generally safe and well tolerated than other antipsychotics, and there were no significant differences in adverse events between the n-3 PUFAs and placebo groups in most studies (30, 50, 51). A subgroup meta-analysis based on ten studies revealed a significantly greater association of treatment with reduced anxiety symptoms in patients receiving at least 2,000 mg/d n-3 PUFAs treatment than in those not receiving it (52).

In our research, n-3 PUFAs supplementation with 2,400 mg/d improve anxiety symptoms faster and may have potential anxiolytic benefits. The potential anxiolytic benefits have been discovered over the past decade (53), and several possible explanations have been proposed. One of the explanations is that the deficiency of n-3 PUFAs and their derivatives in neuronal membranes might induce various behavioral and neuropsychiatric disorders, including anxiety-related behaviors (52). N-3 PUFAs supplementation will change the fatty acid composition of the cell membrane and increase the fluidity of the cell membrane. All of these changes would be beneficial for the relief of anxiety symptoms. Besides, inflammation was consistently found to induce psychotic symptoms, including anxiety (54). And n-3 PUFAs have anti-inflammatory effects which restrain the production of proinflammatory cytokine (55). N-3 PUFAs may help to improve anxiety by regulating the inflammatory factors. Furthermore, proinflammatory cytokines promote the secretion of corticotropin-releasing hormone (CRH), which is the main pathway of the hormonal stress response. CRH also stimulates the amygdala, which is a key brain area for fear and anxiety (56, 57).

An increasing number of dietary or nutrient-based interventions are investigated to be effective in preventing and managing psychiatric disorders (58). However, our research found that supplementation of n-3 PUFAs does not effectively improve depressive symptoms, which is consistent with previously reported studies (59, 60). One possible explanation is that the antidepressant effect of venlafaxine was too strong. So that the relatively weaker effect from n-3 PUFAs could hardly be discovered. Second, n-3 PUFAs supplementation might be much more effective among those with lower n-3 PUFAs levels. Thirdly, CTQ and SSRS might affect the prognostic outcomes of depression. Peng et al. found that mental disorders were associated with higher CTQ scores and lower SSRS scores (34). Childhood experiences, characters, and childhood trauma have all been shown to correlate with the occurrence of anxiety and depressive disorder (61). Moreover, childhood trauma would increase the persistence of comorbidity and chronic disease in patients with anxiety and/or depressive disorders (62) and contribute to the severity of psychopathology (63, 64). The depressed patients who experienced childhood trauma had a poorer clinical course and therapeutic outcomes (65, 66). It was also reported that social support was associated with prognostic outcomes independent of treatment type (67). The interpersonal behaviors of depressed patients usually cause rejection by others, and these rejection experiences in turn increase the severity of depression (68). Patients with mental disorders as lack of security are more likely to experience unsatisfactory social interactions and reduce perceived social support, which in turn will aggravate depression symptoms (68, 69).

Moreover, to confirm this possibility, we explored the relationships between these factors to understand their effect on depressive symptoms. The symptomatic index parameter like SAS, BDI, and EPA levels had significant differences between depressed patients with childhood trauma and patients without childhood trauma. Meanwhile, there were positive correlations between SAS and CTQ score, BDI and CTQ score, negative correlations between SAS and SSRS, BDI and SSRS. These results suggested that Childhood trauma and SSRS have an influence on anxiety, and depression. Besides, the results of ROC found that the combined ROC curve of CTQ and SSRS was more suitable for depressed patients without n-3 PUFAs supplementation to analyze prognostic outcomes, and suggested that the combined influence of CTQ and SSRS should be one of the factors affecting prognostic outcomes for depressed patients without n-3 PUFAs supplementation. These findings supported our previous hypothesis that the combined influence of CTQ and SSRS might be one of the reasons why supplementation with n-3 PUFAs is not effective in improving depressive symptoms. Based on the present results, it was more suitable for depressed patients without n-3 PUFAs supplementation to analyze prognostic outcomes with the combined effect of SSRS and CTQ.

Significantly negative correlations between CTQ score and EPA level at baseline, rather than DHA level, in first-diagnosed, drug-naïve depressed patients were discovered. Childhood trauma has a certain impact on the EPA level of depressed patients. There is growing evidence to support the link between depression and childhood trauma, like the existence of some biological markers that could explain the link including brain-derived neurotrophic factors and other inflammatory markers (70). Similarly, the connection between depression and EPA is also related to biomarkers (71, 72). Which suggested that EPA has a correlation with CTQ score and might be medicated through the effects of some biomarkers.

## Conclusion

The study found that n-3 PUFAs supplementation in favor of venlafaxine alleviated the anxiety symptoms rather than depressive symptoms at the early stage of treatment for first-diagnosed, drug-naïve depressed patients. However, the advantage disappeared in the long-term treatment. Furthermore, childhood abuse and social support are closely related to the clinical and biological characteristics of depression. Both childhood trauma and lack of social support might be predictors of poor prognosis in depression.

## Limitations

Due to withdrawal and non-compliance, and the influence of COVID-19, the follow-up rate was relatively low. The findings cannot be expanded to patients experiencing multiple depressive episodes or who have already received medication. While a placebo for n-3 PUFA was used, there was no placebo comparison for the antidepressant for both groups taking therapeutic doses of venlafaxine. For this case, it is impossible to distinguish the separate treatment efficacy from venlafaxine, placebo, and n-3 PUFAs alone. Furthermore, it is possible that the treatment efficacy of n-3 PUFAs on clinical characteristics may have been overshadowed by venlafaxine.

## Data Availability Statement

The original contributions presented in this study are included in the article/Supplementary Material, further inquiries can be directed to the corresponding author/s.

## Ethics Statement

The studies involving human participants were reviewed and approved by the Ethics Committee of Second Xiangya Hospital. The patients/participants provided their written informed consent to participate in this study.

## Author Contributions

MT and HT designed the experiments. RY, LW, and MT analyzed and interpreted the data and wrote the manuscript. MT, RY, LW, KJ, SC, and CW performed the experiments and collected the data. MT, JC, LW, and JG amended the manuscript. All authors contributed to the article and approved the submitted version.

## Conflict of Interest

The authors declare that the research was conducted in the absence of any commercial or financial relationships that could be construed as a potential conflict of interest.

## Publisher’s Note

All claims expressed in this article are solely those of the authors and do not necessarily represent those of their affiliated organizations, or those of the publisher, the editors and the reviewers. Any product that may be evaluated in this article, or claim that may be made by its manufacturer, is not guaranteed or endorsed by the publisher.
