# Supplementary material for: Omega-3 Polyunsaturated Fatty Acids Supplementation Alleviate Anxiety Rather Than Depressive Symptoms Among First-Diagnosed, Drug-Naïve Major Depressive Disorder Patients: A Randomized Clinical Trial
Source: Front Nutr. 2022 Jul 12;9:876152. doi: 10.3389/fnut.2022.876152 (PMC9315396; doi:10.3389/fnut.2022.876152)
Supplement: Supplementary file 1 [file Data_Sheet_1.docx]

eTable 1. Correlations between symptomatic index and n-3 PUFAs levels in patients with first-diagnosed drug-naïve depression.

|  | n-3 PUFAs levels (r) | | | | |
| --- | --- | --- | --- | --- | --- |
|  | DHA |  | EPA |  | Total n-3 PUFAs |
| **Baseline** |  |  |  |  |  |
| HAMA | 0.221(0.073) |  | 0.011(0.930) |  | 0.131(0.225) |
| SAS | 0.201(0.104) |  | -0.050(0.692) |  | 0.091(0.398) |
| HAMD | 0.072(0.562) |  | -0.103(0.409) |  | 0.096(0.441) |
| BDI | 0.004 (0.975) |  | -0.122 (0.335) |  | 0.013(0.919) |
| **Week4** |  |  |  |  |  |
| HAMA | 0.098(0.492) |  | -0.085(0.558) |  | 0.195(0.166) |
| SAS | -0.038(0.790) |  | 0.011(0.936) |  | 0.031(0.827) |
| HAMD | 0.124(0.392) |  | 0.101(0.489) |  | 0.092(0.517) |
| BDI | 0.068(0.623) |  | 0.106(0.450) |  | 0.021(0.883) |
| **Week12** |  |  |  |  |  |
| HAMA | 0.233(0.177) |  | 0.013(0.941) |  | 0.217(0.203) |
| SAS | 0.014(0.937) |  | 0.042(0.809) |  | -0.021(0.903) |
| HAMD | 0.096(0.583) |  | -0.173(0.321) |  | 0.053(0.757) |
| BDI | 0.094(0.592) |  | -0.042(0.812) |  | 0.083(0.631) |

n-3 PUFAs, Omega-3 polyunsaturated fatty acids; DHA, docosahexaenoic acid; EPA, eicosapentaenoic acid; HAMA, Hamilton anxiety scale; SAS, Self-rating Anxiety Scale; HAMD, Hamilton Depression Scale; BDI, Beck Depression Inventory.

eTable 2. The ROC curve of CTQ for the prognosis of patients with depression.

| Parameter | Area under ROC curve | Standard deviation | P-value | 95% Cl | Cut-off | Sensitivity | Specificity | 1-Specificity |
| --- | --- | --- | --- | --- | --- | --- | --- | --- |
| HAMD |  |  |  |  |  |  |  |  |
| Total effect |  |  |  |  |  |  |  |  |
| 4 weeks | 0.587 | 0.071 | 0.256 | 0.447-0.727 | 59.00 | 0.857 | 0.362 | 0.638 |
| 12 weeks | 0.548 | 0.084 | 0.569 | 0.384-0.711 | 68.00 | 0.920 | 0.250 | 0.750 |
| n-3 PUFAs |  |  |  |  |  |  |  |  |
| 4 weeks | 0.513 | 0.100 | 0.904 | 0.316-0.709 | 43.50 | 0.909 | 0.240 | 0.760 |
| 12 weeks | 0.615 | 0.124 | 0.367 | 0.373-0.857 | 68.00 | 0.923 | 0.333 | 0.667 |
| Placebo |  |  |  |  |  |  |  |  |
| 4 weeks | 0.691 | 0.095 | 0.088 | 0.505-0.877 | 58.50 | 1.000 | 0.455 | 0.545 |
| 12 weeks | 0.508 | 0.115 | 0.942 | 0.283-0.734 | 49.50 | 0.667 | 0.533 | 0.467 |
| HAMA |  |  |  |  |  |  |  |  |
| Total effect |  |  |  |  |  |  |  |  |
| 4 weeks | 0.561 | 0.086 | 0.457 | 0.393-0.728 | 41.50 | 0.353 | 0.824 | 0.176 |
| 12 weeks | 0.563 | 0.083 | 0.453 | 0.401-0.725 | 60.50 | 0.739 | 0.423 | 0.577 |
| n-3 PUFAs |  |  |  |  |  |  |  |  |
| 4 weeks | 0.533 | 0.116 | 0.750 | 0.306-0.760 | 40.00 | 0.333 | 0.958 | 0.042 |
| 12 weeks | 0.653 | 0.119 | 0.224 | 0.420-0.886 | 59.00 | 0.455 | 0.818 | 0.182 |
| Placebo |  |  |  |  |  |  |  |  |
| 4 weeks | 0.648 | 0.114 | 0.299 | 0.426-0.871 | 54.00 | 1.000 | 0.444 | 0.556 |
| 12 weeks | **0.714** | 0.100 | 0.060 | 0.518-0.909 | 59.00 | 0.833 | 0.600 | 0.400 |

eTable 3. The ROC curve of SSRS for the prognosis of patients with depression.

| Parameter | Area under ROC curve | Standard deviation | P-value | 95% Cl | Cut-off | Sensitivity | Specificity | 1-Specificity |
| --- | --- | --- | --- | --- | --- | --- | --- | --- |
| HAMD |  |  |  |  |  |  |  |  |
| Total effect |  |  |  |  |  |  |  |  |
| 4 weeks | 0.629 | 0.078 | 0.089 | 0.477-0.782 | 30.50 | 0.524 | 0.750 | 0.250 |
| 12 weeks | 0.562 | 0.085 | 0.459 | 0.396-0.727 | 27.50 | 0.480 | 0.750 | 0.250 |
| n-3 PUFAs |  |  |  |  |  |  |  |  |
| 4 weeks | 0.609 | 0.111 | 0.303 | 0.391-0.827 | 30.50 | 0.545 | 0.760 | 0.240 |
| 12 weeks | **0.739** | 0.107 | 0.062 | 0.530-0.949 | 31.50 | 0.462 | 1.000 | 0 |
| Placebo |  |  |  |  |  |  |  |  |
| 4 weeks | 0.665 | 0.109 | 0.137 | 0.451-0.879 | 28.50 | 0.700 | 0.652 | 0.348 |
| 12 weeks | **0.789** | 0.093 | 0.011 | 0.606-0.971 | 30.50 | 0.917 | 0.667 | 0.333 |
| HAMA |  |  |  |  |  |  |  |  |
| Total effect |  |  |  |  |  |  |  |  |
| 4 weeks | 0.650 | 0.088 | 0.065 | 0.478-0.821 | 33.50 | 0.412 | 0.885 | 0.115 |
| 12 weeks | 0.564 | 0.084 | 0.446 | 0.399-0.728 | 29.50 | 0.565 | 0.654 | 0.346 |
| n-3 PUFAs |  |  |  |  |  |  |  |  |
| 4 weeks | 0.601 | 0.118 | 0.330 | 0.369-0.832 | 31.50 | 0.500 | 0.875 | 0.125 |
| 12 weeks | 0.517 | 0.130 | 0.896 | 0.262-0.771 | 30.50 | 0.455 | 0.727 | 0.273 |
| Placebo |  |  |  |  |  |  |  |  |
| 4 weeks | **0.764** | 0.095 | 0.063 | 0.577-0.951 | 29.50 | 0.800 | 0.714 | 0.286 |
| 12 weeks | 0.594 | 0.115 | 0.407 | 0.368-0.820 | 26.00 | 0.917 | 0.400 | 0.600 |

eTable 4. The combined ROC curve of CTQ and SSRS for the prognosis of patients with depression.

| Parameter | Area under ROC curve | Standard deviation | P-value | 95% Cl | Cut-off | Sensitivity | Specificity | 1-Specificity |
| --- | --- | --- | --- | --- | --- | --- | --- | --- |
| HAMD |  |  |  |  |  |  |  |  |
| Total effect |  |  |  |  |  |  |  |  |
| 4 weeks | 0.644 | 0.078 | 0.059 | 0.492-0.796 | 0.312 | 0.667 | 0.702 | 0.298 |
| 12 weeks | 0.583 | 0.083 | 0.317 | 0.421-0.746 | 0.538 | 0.520 | 0.708 | 0.292 |
| n-3 PUFAs |  |  |  |  |  |  |  |  |
| 4 weeks | 0.600 | 0.107 | 0.345 | 0.390-0.810 | 0.370 | 0.880 | 0.120 | 0.364 |
| 12 weeks | **0.726** | 0.109 | 0.077 | 0.514-0.939 | 0.748 | 0.462 | 1 | 0 |
| Placebo |  |  |  |  |  |  |  |  |
| 4 weeks | **0.723** | 0.098 | 0.046 | 0.530-0.916 | 0.262 | 0.800 | 0.636 | 0.364 |
| 12 weeks | **0.783** | 0.092 | 0.013 | 0.602-0.965 | 0.394 | 0.917 | 0.733 | 0.267 |
| HAMA |  |  |  |  |  |  |  |  |
| Total effect |  |  |  |  |  |  |  |  |
| 4 weeks | 0.667 | 0.078 | 0.040 | 0.515-0.820 | 0.306 | 0.471 | 0.843 | 0.157 |
| 12 weeks | 0.559 | 0.083 | 0.477 | 0.397-0.722 | 0.444 | 0.739 | 0.423 | 0.577 |
| n-3 PUFAs |  |  |  |  |  |  |  |  |
| 4 weeks | 0.573 | 0.106 | 0.481 | 0.366-0.780 | 0.520 | 0.250 | 1.000 | 0 |
| 12 weeks | 0.645 | 0.122 | 0.250 | 0.406-0.883 | 0.539 | 0.545 | 0.818 | 0.182 |
| Placebo |  |  |  |  |  |  |  |  |
| 4 weeks | **0.763** | 0.100 | 0.065 | 0.567-0.959 | 0.107 | 1 | 0.481 | 0.519 |
| 12 weeks | **0.728** | 0.098 | 0.045 | 0.535-0.920 | 0.369 | 0.600 | 0.400 | 0.385 |
